# Microscopic versus endoscopic transsphenoidal surgery in the Leiden cohort treated for Cushing’s disease: surgical outcome, mortality, and complications

**DOI:** 10.1186/s13023-019-1038-0

**Published:** 2019-03-11

**Authors:** Leonie H. A. Broersen, Femke M. van Haalen, Nienke R. Biermasz, Daniel J. Lobatto, Marco J. T. Verstegen, Wouter R. van Furth, Olaf M. Dekkers, Alberto M. Pereira

**Affiliations:** 1 Department of Medicine, Division of Endocrinology, Leiden University Medical Centre, Albinusdreef 2, 2333 ZA Leiden, The Netherlands; 2 Center for Endocrine Tumors Leiden (CETL), Leiden University Medical Center, Albinusdreef 2, 2333 ZA Leiden, The Netherlands; 3 Department of Neurosurgery, Leiden University Medical Center, Albinusdreef 2, 2333 ZA Leiden, The Netherlands; 4 Department of Clinical Epidemiology, Leiden University Medical Center, Albinusdreef 2, 2333 ZA Leiden, The Netherlands

**Keywords:** Cushing’s disease, Transsphenoidal adenomectomy, Microscopy, Endoscopy, Surgical outcome, Complications

## Abstract

**Background:**

First-choice treatment for Cushing’s disease is transsphenoidal adenomectomy. Since its introduction in the 1970s, many centers have now switched from microscopic to endoscopic surgery. We compared both techniques for the treatment of Cushing’s disease at the Leiden University Medical Center, a European reference center for pituitary diseases.

**Methods:**

Cohort study with inclusion and follow-up of consecutive Cushing’s disease patients primarily treated by transsphenoidal surgery at the Leiden University Medical Center between 1978 and 2016. We compared remission rates (primary endpoint), mortality, and complications between microscopic (performed up to 2005) and endoscopic (performed from 2003 onwards) surgery. Subgroup analyses were performed by tumor size, surgical experience, and preoperative imaging techniques. Additionally, surgeons’ intraoperative findings regarding presence and removal of the adenoma were related to surgical outcome.

**Results:**

Of 137 included patients, 87 were treated microscopically and 50 endoscopically. Three months after microscopic surgery, 74 patients (86%) were in remission. Five-year recurrence-free survival was 89% (95% confidence interval [CI]: 82–96%), and ten-year recurrence free survival was 84% (95% CI: 75–93%). After endoscopic surgery, 39 patients (83%) were in remission. Both five-year and ten-year recurrence-free survival were 71% (95% CI: 55–87%). Hazard ratio for recurrence was 0.47 (95% CI: 0.19–1.14), and for mortality 2.79 (95% CI: 0.35–22.51), for microscopic versus endoscopic surgery. No learning curve was found for endoscopy, nor an influence of preoperative imaging technique for microscopy. In addition, we did not find a clear relation between the surgeons’ intraoperative findings and surgical outcomes.

**Conclusions:**

This study did not identify a clear advantage of microscopic or endoscopic transsphenoidal surgery for the treatment of Cushing’s disease based on clinical outcome. The transition to endoscopic surgery at our center was not accompanied by transient worsening of outcomes, which may be reassuring for those considering transitioning.

## Background

Cushing’s disease is caused by an adrenocorticotropic hormone (ACTH)-secreting pituitary adenoma, resulting in endogenous glucocorticoid excess. The incidence is estimated to be 1.2–1.7 per million each year [1]. Glucocorticoid excess causes osteoporosis, central obesity, insulin resistance, dyslipidemia, hypertension, hypercoagulability, and neuropsychiatric disorders [2, 3]. First-choice treatment remains transsphenoidal pituitary surgery, selectively removing the corticotroph adenoma [4, 5]. Despite biochemical cure, mortality risk remains increased in Cushing’s disease patients [6].

Two main techniques have been used for transsphenoidal pituitary surgery: microscopic and endoscopic surgery. The endoscope has also been used to confirm findings during microscopic surgery, which is called endoscopy assisted surgery. Microscopic surgery used to be the established method for transsphenoidal surgery. However, since 1992, when the endoscope was first used as a tool to assist in microscopic surgery for a pituitary adenoma [7], many centers have switched to a full endoscopic technique, which was first described in 1997 for treatment of Cushing’s disease [8]. However, proper evaluation of outcomes in a single center has yet to be performed. A limited number of small cohort studies (13–25 patients per study group) have compared both the microscopic and endoscopic techniques for treatment of Cushing’s disease performed in the same center [9–12]. None showed a significant difference in remission rate or surgical morbidity between microscopic and (transition to) endoscopic surgery. Additionally, significant differences in remission rates based on tumor size could not be determined [9]. Several systematic reviews concerning transsphenoidal microscopic versus endoscopic surgery have evaluated outcomes for all pituitary adenomas and found comparable results for remission and recurrence rates, and inconclusive results regarding complication rates [13–15]. Theoretically, endoscopic surgery has the advantage of achieving better tumor visualization, mainly in laterally invasive or large tumors. This increased visibility enables improved resectability, leading to potentially higher remission rates. However, these advantages have also led to a higher risk of cerebrospinal fluid (CSF) leakage, most likely due to a more aggressive surgical approach in an attempt to obtain complete adenoma removal [16]. Several studies have reported on a learning curve for the endoscopic technique [17–20], showing that outcomes improve with increased surgical experience, which possibly leads to biased results in series when comparing between years of experience in the microscopic surgical technique and the newer endoscopic technique.

Due to the lack of comparability of available studies, we present our long-term data on transsphenoidal surgery for Cushing’s disease, reporting on both surgical outcome, with as primary endpoint remission rate, as well as complication rates for both techniques. To date, there are unresolved questions regarding long-term remission rates and surgical morbidity after both treatments, overall as well as in relation to tumor size. Furthermore, differences in long-term mortality, endocrinological complications, and morbidity between the two surgical techniques need to be explored to be able to properly evaluate both treatment options. Finally, in order to establish the predictive value of intraoperative findings, it is important to relate the surgeons’ intraoperative findings regarding presence and removal of the adenoma to surgical outcome.

### Study aims

The primary study aim was to compare microscopic and endoscopic transsphenoidal surgery for Cushing’s disease regarding: 1) Surgical outcome (remission rate = primary endpoint, recurrence, and hydrocortisone dependency), 2) Long-term mortality risk, and 3) Short- and long-term surgical and endocrinological complications. Secondary study aims were to stratify these results based on 1) Tumor size and 2) Surgical experience for endoscopy, and 3) To relate surgeons’ intraoperative findings regarding presence and removal of the adenoma to surgical outcome.

## Methods

### Study population

Consecutive Cushing’s disease patients primarily treated with transsphenoidal surgery at the tertiary referral center Leiden University Medical Center, the coordinating center of the newly established European Reference Network for rare Endocrine conditions, and a European reference center for pituitary diseases, were included in this cohort study. Inclusion started in 1978, when the first transsphenoidal operation in the Netherlands was performed in Leiden. Microscopic surgery was performed from 1978 to 2005, whereas endoscopic surgery has been performed since 2003. Patients were included if surgery was performed before January 1st 2017. Patients were excluded if they had undergone a unilateral adrenalectomy and/or pituitary irradiation prior to transsphenoidal surgery. There were no restrictions in presurgical medical treatment with cortisol lowering agents or in adjuvant therapy in case of persistent disease or for recurrence of disease.

The diagnosis of Cushing’s disease was made based both on clinical grounds and biochemical tests: increased morning serum cortisol (> 500 nmol/L), increased 24-h urinary free cortisol (UFC) excretion (> 220 nmol until 2010, after which the cut-off level was > 150 nmol), increased midnight salivary cortisol excretion (> 5.7 nmol/L, measured since 2004), insufficient suppression of morning serum cortisol after low-dose dexamethasone (1 mg in the evening), as well as a non-suppressed ACTH. Until 1988, urinary 17-KgS was measured in some patients instead of UFC (increased if > 16 mg/24 h for women and > 22 mg/24 h for men). All patients underwent pituitary imaging either by computed tomography (CT, until 1994, and one patient in 2009 due to a contraindication for magnetic resonance imaging [MRI]), or MRI. Three patients who were operated before 1980 underwent imaging by sellar planigraphy, with or without basal cisternography (X-ray). If pituitary imaging yielded inconclusive results, bilateral simultaneous sampling of the inferior petrosal sinuses (IPSS) was performed. Subsequently, pituitary surgery with exploration of the sella was performed when the results of IPSS were consistent with a pituitary source of ACTH overproduction. Otherwise, imaging studies (CT of the thorax and octreotide or gallium DOTATATE PET scan) were performed to identify an ectopic source of ACTH overproduction. In case no ectopic ACTH-producing tumor was found, diagnostic tests were repeated after a period of watchful waiting, and surgical treatment was performed only after imaging or IPSS indicated a pituitary adenoma.

### Interventions and postoperative evaluation

The primary treatment was either microscopic or endoscopic transsphenoidal pituitary adenomectomy for all included patients. A direct transnasal transsphenoidal microscopic approach was performed solely until 2002. From 2003 onwards, the endoscopic procedure was introduced in our center for the surgical treatment of pituitary tumors, including Cushing’s disease. At first, an endoscopic assisted approach was used to confirm findings of the microscope. In this study, endoscopic assisted surgery was analysed in the study group of microscopic surgery, because the surgery was primarily performed using the microscope, which was therefore the main technique to be considered for treatment success and complication rates. From 2006 onwards, purely endoscopic transnasal transsphenoidal approaches were the only surgical treatment modality. All surgical procedures were performed by three dedicated pituitary neurosurgeons. Postoperative biochemical evaluation was always performed within two weeks. Three to six months postoperatively, remission was defined based on both clinical criteria (dependency on hydrocortisone replacement, or hydrocortisone independency without any biochemical signs of hypercortisolism and regression of clinical signs) and biochemical criteria (morning cortisol suppression after 1 mg dexamethasone to below 50 nmol/L and normal 24-h UFC excretion or midnight salivary cortisol excretion in two consecutive samples, if not on hydrocortisone replacement) [21]. Persistent Cushing’s disease was defined as the absence of remission after first surgery. Disease recurrence was defined as biochemical recurrence according to the aforementioned criteria, and re-occurrence of clinical signs, after a period of remission of at least three months.

### Endpoints and follow-up

Study endpoints were surgical outcomes, mortality, and short- and long-term morbidity. Surgical outcomes considered were the following: 1) Remission rate (primary endpoint), 2) Persistent disease, 3) Recurrent disease, and 4) Hydrocortisone dependency three months after surgery (divided in three categories: a) Absolute deficiency [insufficient cortisol response to corticotropin-releasing hormone (CRH)-test or equivalent], b) Hydrocortisone to treat withdrawal symptoms despite normal cortisol response to CRH stimulation, and c) Pragmatic hydrocortisone replacement without stimulation test).

Short-term morbidity (≤3 month after first surgery): 1) Postoperative CSF-leakage, 2) Bacterial meningitis (or start of antibiotic treatment due to suspicion of meningitis), 3) All intra- and postoperative bleedings (considered were internal carotid artery injury, epistaxis, severe venous blood loss), 4) Severe bleeding (requiring surgical intervention or described as severe in patient file), 5) Syndrome of inappropriate antidiuretic hormone secretion (Na < 135 mmol/L), 6) Diabetes insipidus (transient, requiring medication at least once), 7) Anterior pituitary deficiency other than ACTH requiring medication, 8) Corticosteroid withdrawal syndrome (complaints and/or requirement of increase in hydrocortisone replacement dose) [22], and 9) Cardiovascular morbidity (thrombosis, pulmonary embolism, cerebrovascular accident, transient ischemic attack, and myocardial ischemia).

Long-term morbidity (> 3 months after first surgery): 1) Anterior pituitary deficiency other than ACTH requiring medication (measured one year after surgery), 2) Hydrocortisone dependency for > 3 years (for patients with > 3 years follow-up), 3) Diabetes insipidus (permanent: > 3 months), 4) Cardiovascular morbidity, 5) Hypertension (de novo as well as persisting after surgery), 6) Diabetes mellitus (de novo as well as persisting after surgery), and 7) Neuropsychiatric morbidity (complaints/symptoms as well as consultation of psychologist or psychiatrist).

Follow-up was defined as time between date of surgery and death, loss to follow-up, or December 31st 2016, whichever came first. For time-to-event analyses, follow-up was defined as time between date of surgery and outcome event (mortality or recurrence), death, loss to follow-up, or December 31st 2016, whichever came first. Presurgical information was collected regarding diagnosis (including Cushing’s syndrome severity index score [CSI score] [23]), comorbidities (hypertension, diabetes mellitus, dyslipidemia), and medical treatment prior to surgery. In case of loss to follow-up, data collection was completed to our best ability by contacting both patient and current health care provider.

Tumor characteristics: tumor size on preoperative imaging divided tumors into microadenomas (≤10 mm) and macroadenomas (> 10 mm). Giant adenomas (> 3 cm) were not analysed separately. Intraoperative findings were classified into three categories: complete adenomectomy (surgeon reported complete removal of the adenoma), (at least) partial adenomectomy (surgeon reported partial removal of the adenoma, but is uncertain whether there is residual tumor), and residual tumor (surgeon is certain there is residual tumor).

### Risk of bias

Consecutive patients were included in this study to minimize selection bias, although selection could still be a cause of bias in our data. Patients were assigned to a treatment method based on the availability of the treatment method in our center at time of inclusion, although this does not guarantee comparability of both study groups, e.g. due to time trends. To assess potential differences through time, we plotted percentage macroadenomas per period of five calendar years. Selective loss to follow-up could have led to selection bias [24], although this was minimized by our attempts at complete data collection, reducing the number of patients lost to follow-up by 21. Selective referral of patients from other centers, mainly patients with (difficult and/or invasive) macroadenomas, could potentially have led to selection bias if referral was different for microscopy and endoscopy. Interventions were unlikely to be misclassified. Confounding is a potential source of bias and was assessed by comparing baseline patient characteristics between the study groups. Furthermore, follow-up time was assessed as a potential source of bias.

### Statistical analysis

To describe homogeneity between the surgical groups as well as between microadenomas and macroadenomas, various parameters were analysed by bivariate test. Contingency tables were presented, comparing characteristics of microscopic and endoscopic surgery. A table was prepared with short- and long-term morbidity according to tumor size, comparing microscopic to endoscopic surgery. All variables in Tables 1 and 2 were compared between the two surgical techniques for the entire cohort as well as for microadenomas and macroadenomas separately as this is considered an effect modifier. We used the unpaired T-test for continuous outcomes, and the two-sample test of proportions for categorical variables (in case of > 2 categories, we performed this test separately per category to calculate the difference between the groups). Additionally, the Chi-squared test was used to calculate one *p*-value for categorical variables with > 2 categories. If data were missing for ≥5% of patients per parameter, this was marked in the tables. An “as treated” analysis was performed, excluding patients with missing data for confounding variables from the adjusted analyses.

**Table 1:** demographic characteristics

	Microscopic trans-sphenoidal adenomectomy		Endoscopic transsphenoidal adenomectomy		Tested difference (95% CI; *p*-value)	Tested difference (95% CI; p-value) (Microadenoma only)	Tested difference (95% CI; p-value) (Macroadenoma only)
	N	%	N	%			
*Total*	87	100.0	50	100.0		Sample size:103	Sample size:34
Age at diagnosis, years*	38.9	15.4	44.4	15.1	5.5 (0.1 to 10.9; *p* = 0.045)	3.4 (−2.6 to 9.3; *p* = 0.263)	9.0 (−3.5 to 21.4; *p* = 0.152)
*Sex (female)*	69	79.3	34	68.0	11.3 (−4.2 to 26.8; *p* = 0.140)	7.7 (− 10.4 to 25.8; *p* = 0.391)	20.8 (−8.5 to 50.1; *p* = 0.170)
*Calendar year of transsphenoidal surgery°*	1992	1978–2005	2011	2003–2016	19.2 (17.1 to 21.2; *p* = 0.000)	20.4 (18.1 to 22.6; *p* = 0.000)	14.7 (10.1 to 19.3; p = 0.000)
*Duration of follow-up (years)* ^*$*^	16.9	11.7–26.7	4.7	2.0–7.4	13.4 (11.0 to 15.8; p = 0.000)	14.0 (11.2 to 17.7; p = 0.000)	11.3 (6.9 to 15.6; p = 0.000)
Comorbidities at diagnosis							
*Hypertension*	64	73.6	38	76.0	2.4 (− 12.6 to 17.4; *p* = 0.756)	3.3 (−15.2 to 21.8; *p* = 0.723)	15.3 (− 11.0 to 41.6; *p* = 0.271)
*Diabetes mellitus*	14	16.1	15	30.0	13.9 (−1.0 to 28.8; *p* = 0.055)	13.5 (−4.1 to 31.1; *p* = 0.110)	14.5 (−14.0 to 43.0; *p* = 0.320)
*Dyslipidemia*	8	9.1	8	16.0	6.9 (− 4.9 to 18.7; *p* = 0.225)	3.0 (−8.0 to 14.0; *p* = 0.569)	9.0 (−20.7 to 38.7; *p* = 0.553)
Cushing’s syndrome Severity Index score*	6.9	2.3	6.8	2.8	−0.10 (−0.98 to 0.79; *p* = 0.832)	0.09 (−1.00 to 1.17; *p* = 0.877)	− 0.72 (−2.31 to 0.86; *p* = 0.357)
*0–4*	13	15.1	10	20.0			
*5–8*	53	61.6	27	54.0			
*9–16*	20	23.2	13	26.0			
Tumor size					11.3 (−4.2 to 26.8; p = 0.140)	–	–
*Microadenoma*	69	79.3	34	68.0			
*Macroadenoma Cavernous sinus invasion*	185	20.7 27.8	16 8	32.0 50.0			
Referral by other neurosurgeon or university medical center	4	4.6	6	12.0	7.4 (−2.6 to 17.4; *p* = 0.109)	3.0 (−5.9 to 11.9; *p* = 0.459)	13.9 (−11.8 to 39.6; *p* = 0.389)
Medical treatment prior to surgery	57	65.5	47	94.0	28.5 (16.5 to 40.5; p = 0.000)	34.8 (23.6 to 46.0; p = 0.000)	14.6 (−14.4 to 43.6; *p* = 0.335)
*-Metyrapone*	8	9.2	16	32.0			
*-Ketoconazole*	51	58.6	32	64.0			
*-Pasireotide*	0	0.0	2	4.0			

*mean + standard deviation, °mean + range, ^$^median + interquartile range; CI = confidence interval

**Table 2:** diagnostic strategy and results

	Microscopic trans-sphenoidal adenomectomy		Endoscopic transsphenoidal adenomectomy		Tested difference (95% CI; p-value)	Tested difference (95% CI; p-value) (Microadenoma only)	Tested difference (95% CI; p-value) (Macroadenoma only)
	N	%	N	%			
*Inferior petrosal sinus sampling*	30	34.5	13	26.0	8.5 (−7.2 to 24.2; *p* = 0.302)	2.4 (−17.6 to 22.4; *p* = 0.815)	^
*Radiologic imaging*					p = 0.000	p = 0.000	*p* = 0.003
*-CT or X-ray*	27	31.0	1	2.0	29.0 (18.5 to 39.5)	31.9 (19.3 to 44.5)	16.7 (−0.5 to 33.9)
*-MRI 1.5 Tesla*	60	69.0	18	36.0	33.0 (16.5 to 49.5)	38.7 (20.1 to 57.3)	27.0 (−2.8 to 56.8)
*-MRI 3 Tesla*	0	0.0	31	62.0	62.0 (48.5 to 75.4)	70.6 (55.3 to 85.9)	43.8 (19.5 to 68.1)
*Radiology results*					*p* = 0.144	*p* = 0.359	*p* = 0.339
*-Adenoma*	56	64.4	40	80.0	15.6 (0.6 to 30.6)	14.1 (−5.2 to 33.4)	5.6 (−5.0 to 16.2)
*-No adenoma*	24	27.6	7	14.0	13.6 (1.6 to 27.0)	12.7 (−4.9 to 30.3)	5.6 (− 5.0 to 16.2)
*-Inconclusive*	7	8.0	3	6.0	2.0 (−6.7 to 10.7)	1.3 (−10.6 to 13.2)	#
*Histology results*					*p* = 0.784	*p* = 0.940	p = 0.339
*-Adenoma*	67	77.9	41	82.0	4.1 (−9.7 to 17.9)	0.0 (−18.2 to 18.2)	5.6 (−5.0 to 16.2)
*-No adenoma*	16	18.6	7	14.0	4.6 (−8.1 to 17.3)	1.5 (−15.3 to 183)	5.6 (−5.0 to 16.2)
*-Inconclusive*	3	3.5	2	4.0	0.5 (−6.2 to 7.2)	1.5 (−7.8 to 10.8)	#
*Immunohistochemistry results: ACTH-positive*	57	66.3	45	90.0	23.7 (10.7 to 36.7; *p* = 0.002)	23.5 (6.9 to 40.1; *p* = 0.015)	16.7 (−0.5 to 33.9; *p* = 0.087)
*Surgeon’s intraoperative findings*					*p* = 0.123	*p* = 0.231	*p* = 0.447
*-Complete adenomectomy*	48^$^	58.5	25	50.0	8.5 (−9.0 to 26.0)	9.8 (−10.9 to 30.5)	8.4 (−24.9 to 41.7)
*-(At least) partial adenomectomy*	15^$^	18.3	18	36.0	17.7 (2.0 to 33.4)	18.2 (−0.8 to 37.2)	19.5 (−7.9 to 46.9)
*-Residual tumor*	4^$^	4.9	2	4.0	0.9 (−6.3 to 8.1)	1.5 (−1.5 to 4.5)	5.1 (− 19.2 to 29.4)
*-No tumor identified*	15^$^	18.3	5	10.0	8.3 (−3.5 to 20.1)	6.8 (−8.7 to 22.3)	5.9 (−5.3 to 17.1)

ACTH = adrenocorticotropic hormone, CI = confidence interval, CT = computed tomography, MRI = magnetic resonance imaging

^Inferior petrosal sinus sampling was considered only for patients with uncertain pituitary tumor, and therefore never in macroadenomas, ^$^Data were missing for ≥5% of patients, #No patients with a macroadenoma with this result, therefore, no analysis could be performed

To compare mortality and remission rates time-to-event analyses were performed. Kaplan-Meier curves were constructed to visualize overall survival and recurrence-free survival. Separate curves were constructed for microadenomas and macroadenomas, thereby including tumor size as stratification factor, and separate curves were constructed stratified by surgeons’ intraoperative findings. As sensitivity analyses, time-to-event curves were constructed comparing the early and late years of all endoscopic operations to assess a potential learning curve, and separate curves were constructed comparing microscopic operations after CT or X-ray imaging versus after MRI to check whether preoperative imaging quality influenced surgical success.

Cox proportional hazard regression analyses were performed to provide hazard ratios and to adjust for potential confounders (results presented in text and Table 3). To take into account different follow-up times, we performed recurrence-free survival analyses also separately with inclusion of only the first 5 years of follow-up. We considered the following variables as potential confounders based on literature and known biological pathways: age at diagnosis, sex, hypertension at diagnosis, diabetes mellitus at diagnosis, dyslipidemia at diagnosis, CSI score, tumor size, and prior medical treatment. After comparison of these potential confounders, we included age at diagnosis and tumor size as confounders in all our Cox analyses, unless otherwise specified.

**Table 3:** short- and long-term morbidity

	Hazard ratio (95% CI; p-value)^#^, unadjusted	Hazard ratio (95% CI; p-value)^#^, adjusted for age at diagnosis and tumor size (microadenoma or macroadenoma)
Surgical outcome		
*-Persistent disease*	0.82 (0.34 to 2.01; *p* = 0.663)	0.88 (0.35 to 2.21; *p* = 0.789)
*-Recurrent disease*	0.47 (0.19 to 1.14; *p* = 0.093)	0.40 (0.16 to 1.01; *p* = 0.052)
*-Hydrocortisone dependency* *- Absolute deficiency* - Normal cortisol response* *- Pragmatic replacement* ^*$*^	1.11 (0.73 to 1.68; *p* = 0.625) 1.06 (0.69 to 1.62; *p* = 0.793)	1.04 (0.68 to 1.60; *p* = 0.845) 0.97 (0.63 to 1.50; *p* = 0.888)
*-Repeat surgery* *-Remission after first repeat surgery*	0.30 (0.13 to 0.70; *p* = 0.005) 0.21 (0.04 to 1.13; *p* = 0.069)	0.27 (0.11 to 0.64; *p* = 0.003) 0.17 (0.02 to 1.16; *p* = 0.070)
*Short term morbidity: ≤3 month after first surgery*		
*-Cerebrospinal fluid leakage*	°	°
*-Meningitis*	°	°
*-Bleeding, all*	0.56 (0.22 to 1.41; *p* = 0.221)	0.56 (0.22 to 1.44; *p* = 0.227)
*-Bleeding, severe*	°	°
*-Syndrome of inappropriate antidiuretic hormone release*	0.32 (0.09 to 1.09; p = 0.069)	0.29 (0.08 to 1.02; *p* = 0.054)^a^
*-Diabetes insipidus*	1.04 (0.54 to 1.99; *p* = 0.904)	0.96 (0.49 to 1.86; *p* = 0.901)
*-Anterior pituitary deficiency* *- one axis* *- two axes* *- three axes*	0.76 (0.37 to 1.54; *p* = 0.444) 0.85 (0.37 to 1.96; *p* = 0.704) 0.41 (0.09 to 1.83; *p* = 0.243)	1.06 (0.51 to 2.22; *p* = 0.870) 1.18 (0.50 to 2.79; *p* = 0.708) 0.52 (0.11 to 2.45; *p* = 0.409)
*-Corticosteroid withdrawal syndrome*	0.39 (0.21 to 0.72; p = 0.003)	0.38 (0.20 to 0.72; p = 0.003)
*-Cardiovascular morbidity*	°	°
*Long term morbidity: > 3 months after first surgery*		
*-Anterior pituitary deficiency after 1 year* *- one axis* *- two axes* *- three axes*	0.79 (0.42 to 1.52; *p* = 0.488) 0.83 (0.38 to 1.83; *p* = 0.642) 0.65 (0.17 to 2.41; *p* = 0.517)	1.03 (0.52 to 2.01; *p* = 0.056) 1.10 (0.48 to 2.48; *p* = 0.827) 0.74 (0.19 to 2.89; p = 0.663)
*-Hydrocortisone dependency > 3 years*	1.05 (0.56 to 1.96; *p* = 0.878)	1.11 (0.59 to 2.10; *p* = 0.742)
*-Diabetes insipidus > 3 months*	1.71 (0.68 to 4.29; *p* = 0.251)	1.84 (0.72 to 4.69; *p* = 0.204)
*-Cardiovascular morbidity*	0.37 (0.12 to 1.16; *p* = 0.088)	0.43 (0.13 to 1.38; *p* = 0.155)
*-Hypertension*	0.85 (0.51 to 1.41; *p* = 0.529)	0.92 (0.53 to 1.58; *p* = 0.749)
*-Diabetes mellitus*	0.53 (0.22 to 1.29; *p* = 0.160)	0.56 (0.23 to 1.36; *p* = 0.198)
*-Neuropsychiatric morbidity*	0.14 (0.03 to 0.68; *p* = 0.015)	0.15 (0.03 to 0.76; *p* = 0.022)

CI = confidence interval

^#^Reference group for hazard ratios was endoscopic surgery

^a^Adjusted for age at diagnosis only

*Hydrocortisone for symptoms despite normal cortisol response to CRH stimulation. °Insufficient data for analysis

^$^Pragmatic hydrocortisone replacement (without stimulation test)

Statistical analyses were performed with IBM SPSS Statistics 23.0 (IBM Corp, Armonk, NY, USA) and with Stata 14.2 (Stata Corp., College Station, TX, USA) for the two-sample test of proportions (command: prtesti) to calculate the difference between two proportions with 95% confidence interval (CI), as this was not provided by SPSS. All patients gave informed consent to use their data for scientific research and permission from the ethical committee (MEC) in the LUMC was granted. The Strengthening the Reporting of Observational Studies in Epidemiology (STROBE) guidelines were used for reporting [25].

## Results

### Study population (Fig. 1, and Table 1)

In total, 137 patients were included, of whom 87 (79.3% female, mean age 38.9 [range 12–80] years) underwent microscopic and 50 (68% female, mean age 44.4 [range 10–73] years) endoscopic surgery. Macroadenomas were relatively more common in the endoscopic group (32.0% versus 20.7% in the microscopy group), and preoperative medical treatment was more common in patients with endoscopic surgery (94.0% versus 65.5% in the microscopy group), in line with the changing treatment strategies over time. Fifteen patients were lost to follow-up (all in the microscopic surgery group after an average of 101 months, range: 3–261 months). Histopathology confirmed a corticotroph adenoma in nineteen patients with negative imaging, and was inconclusive in one patient. See Table 2 for a detailed description of diagnostic strategy and results.

**Fig. 1:**
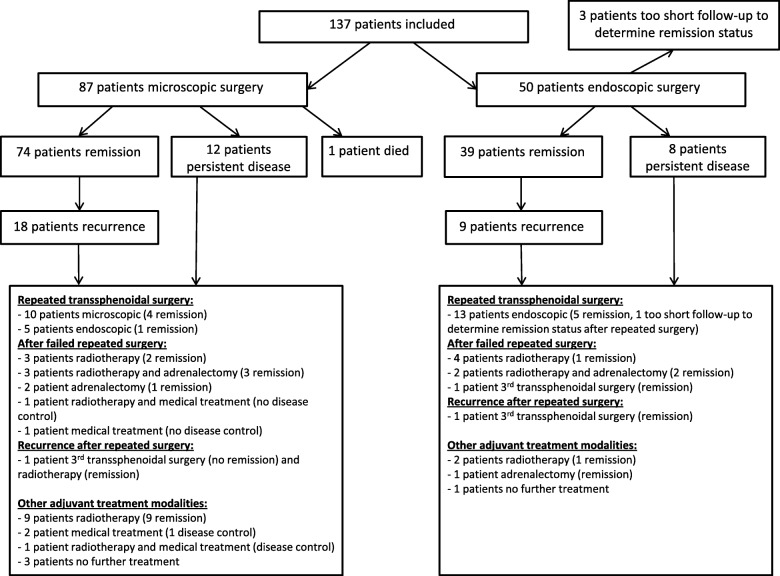
Flow chart of treatment and remission status

### Surgical outcome and recurrence-free survival

Three months after microscopic surgery, 74 of 86 patients (86.0%) were in remission, of whom 67 patients (77.9%) were hydrocortisone dependent. One patient died three days after microscopic surgery (cause not identified). The five-year recurrence-free survival rate for the patients in remission after surgery was 89% (95% CI: 82–96%), and the ten-year recurrence free survival rate was 84% (95% CI: 75–93%).

Three months after endoscopic surgery, 39 of 47 patients (83.0%) were in remission, of whom 33 patients (70.2%) were hydrocortisone dependent. Both the five-year and ten-year recurrence-free survival rates were 71% (95% CI: 55–87%) for the patients in remission after surgery. All patients with negative pathology were in remission at the end of follow-up, except for one patient. Patients with negative pathology achieved remission after (repeat) transsphenoidal surgery or pituitary radiotherapy, and in three cases after adjuvant bilateral adrenalectomy.

**Fig. 2:**
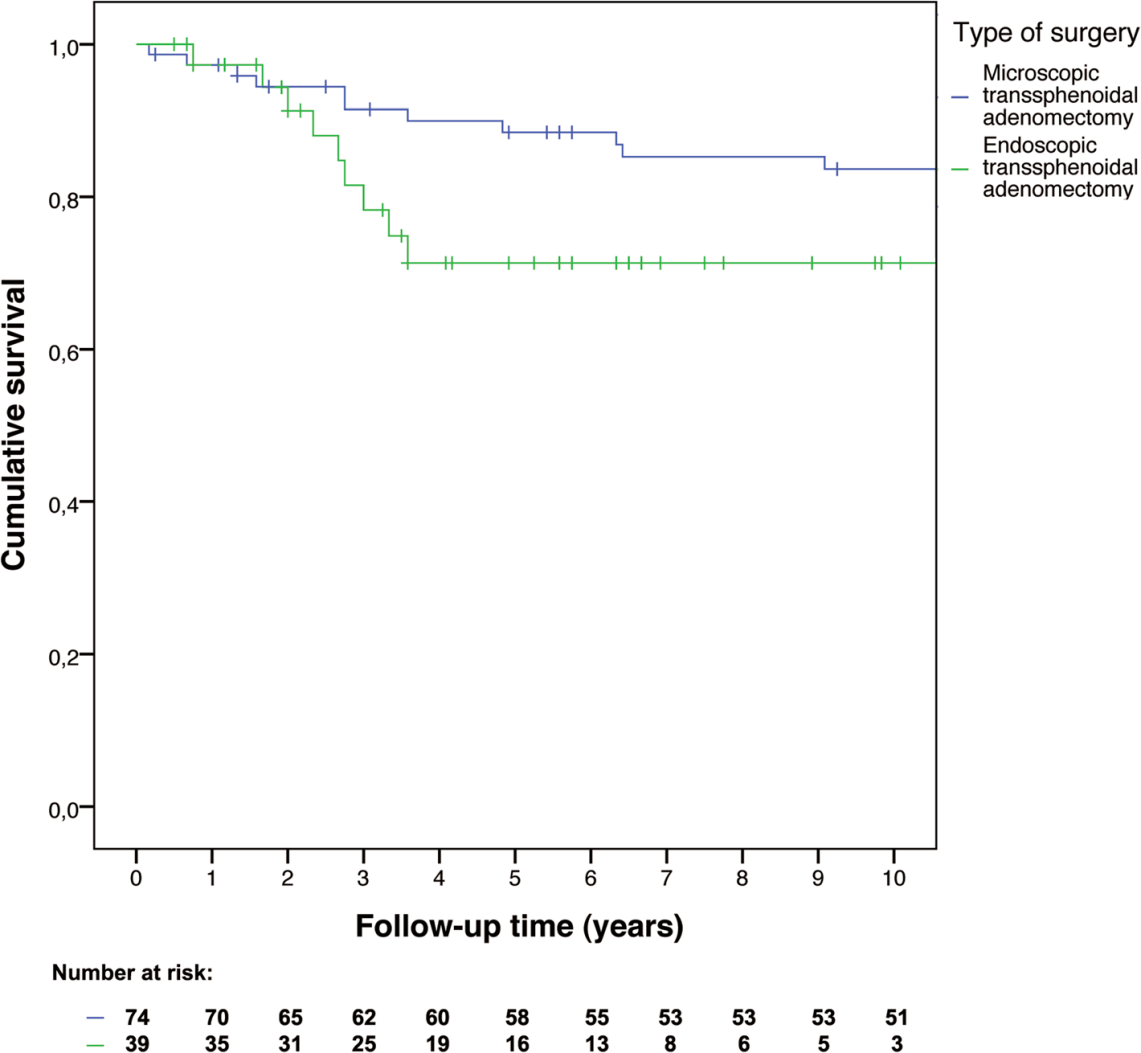
Recurrence-free survival after microscopic versus endoscopic transsphenoidal surgery

**Fig. 3:**
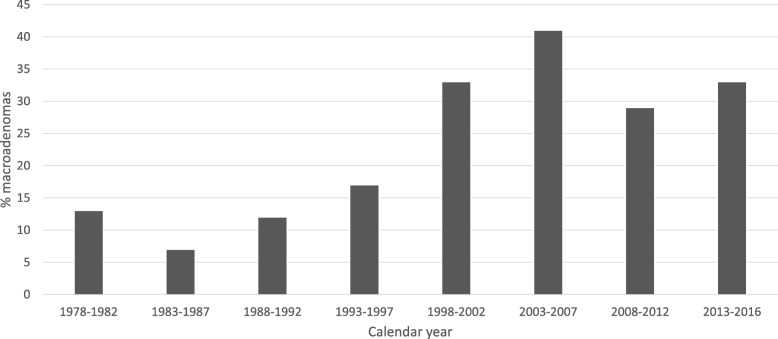
Percentage of macroadenomas operated during the study period

**Fig. 4:**
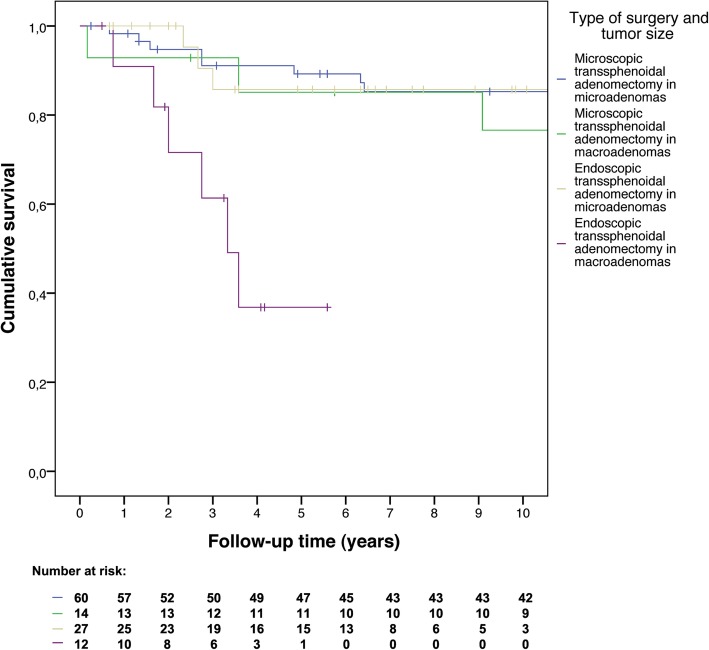
Recurrence-free survival per tumor size after microscopic versus endoscopic transsphenoidal surgery

Recurrence occurred less often in patients with microscopic surgery (hazard ratio 0.47, 95% CI: 0.19–1.14), see Fig. 2. The hazard ratio was 0.38 (95% CI: 0.15–0.99) if only the first 5 years of follow-up were considered. After adjustment, the hazard ratio was 0.40 (95% CI: 0.16–1.01). As percentage macroadenomas changed over time (Fig. 3), separate analyses for microadenomas and macroadenomas were performed. For microadenomas the risk of recurrence was similar for the two techniques: hazard ratio 0.99 (95% CI: 0.26–3.74), or hazard ratio 0.79 (95% CI: 0.20–3.16) for the first 5 years of follow-up, which was similar after adjustment for age at diagnosis. If only macroadenomas were considered, the hazard ratio was 0.18 (95% CI: 0.04–0.91, *p* = 0.038), which was exactly the same for the first 5 years of follow-up, and 0.14 (95% CI: 0.03–0.77; *p* = 0.023) after adjustment for age at diagnosis, see Fig. 4. When patients with cavernous sinus invasion were excluded from analysis, the hazard ratio for macroadenomas remained similar, also after adjustment for age at diagnosis.

**Fig. 5:**
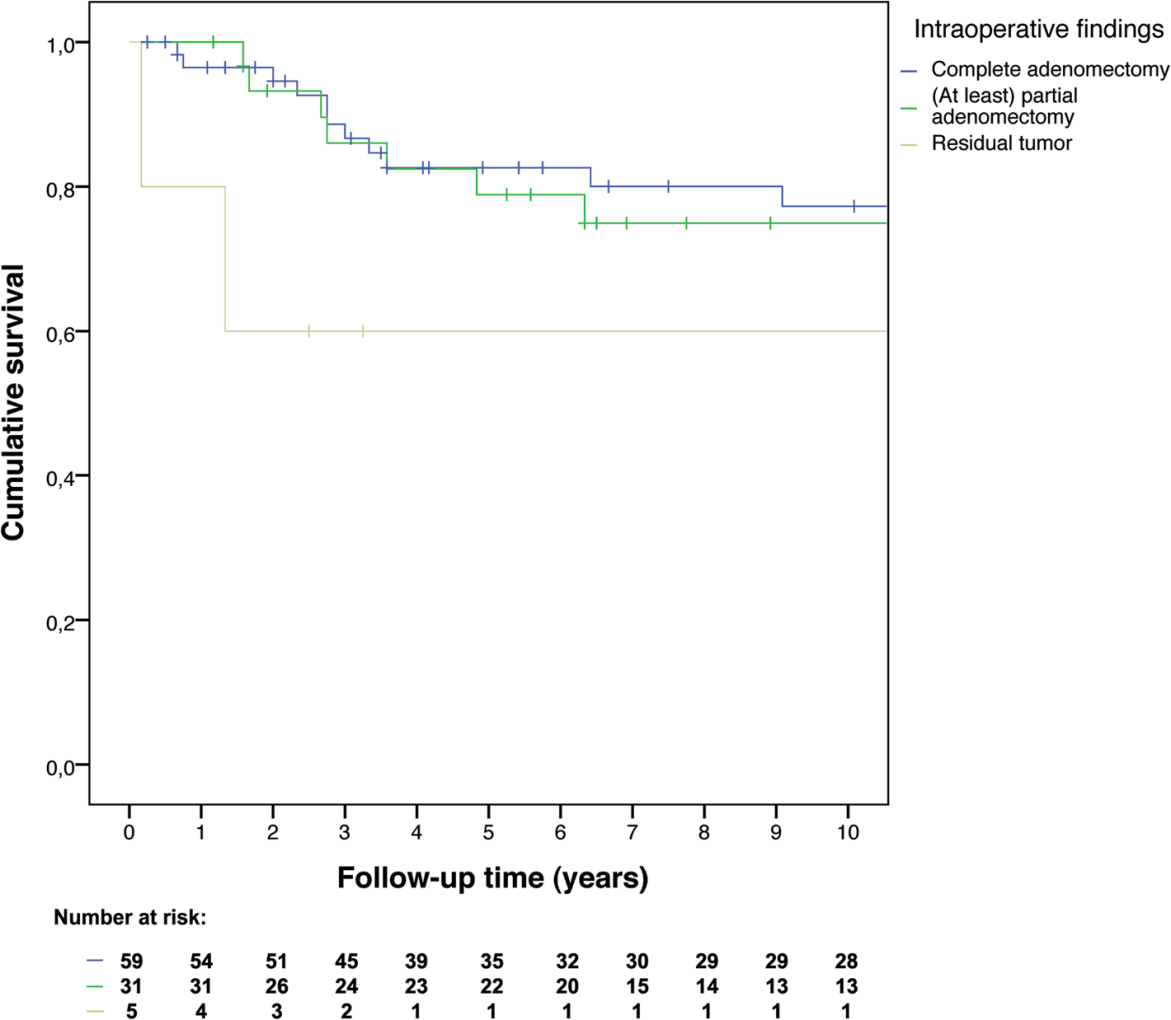
Recurrence-free survival according to surgeons’ intraoperative findings

**Fig. 6:**
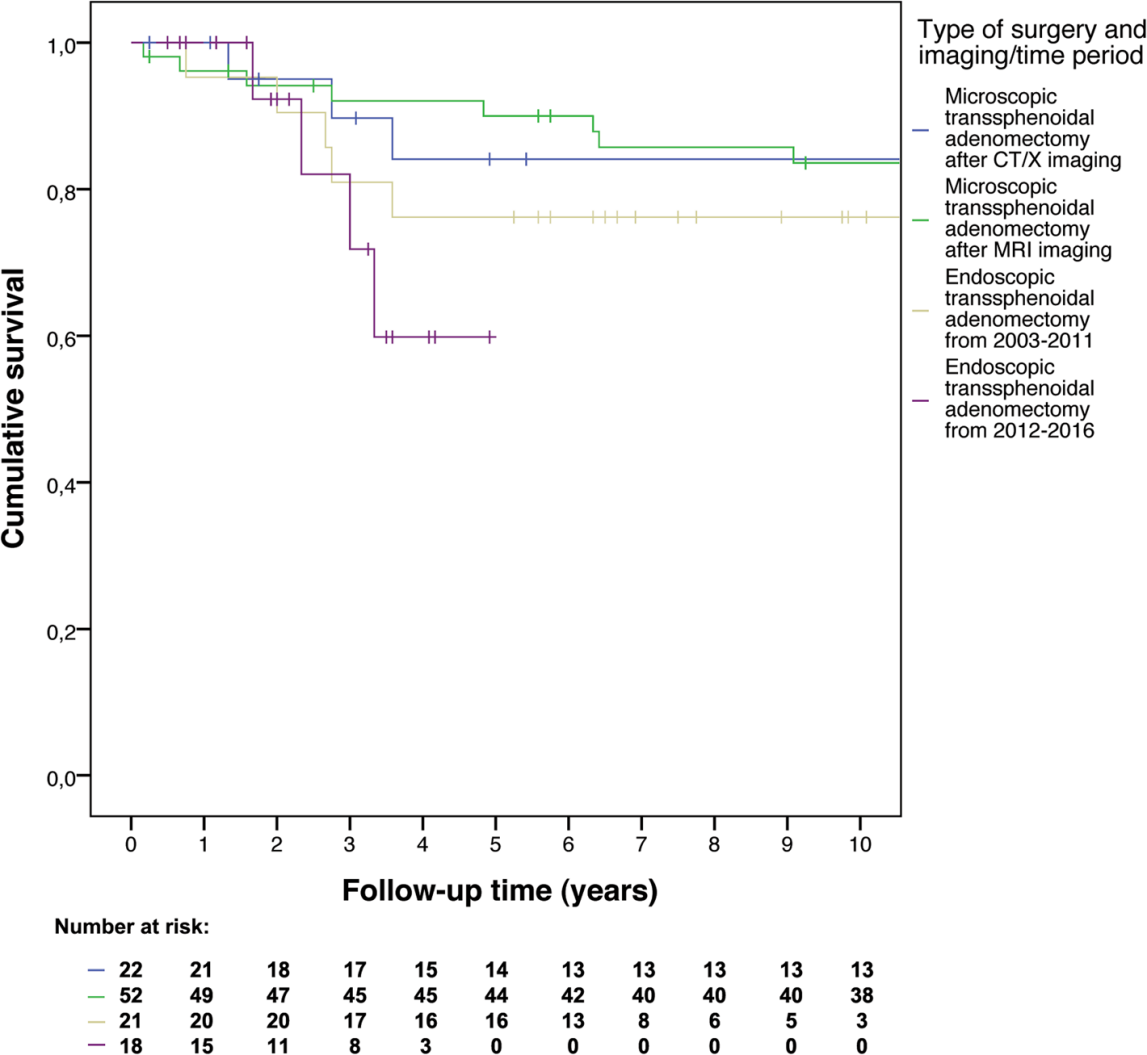
Recurrence-free survival according to imaging and time of surgery

Surgeons’ intraoperative findings were not clearly related to recurrence-free survival (see Fig. 5). Hazard ratios compared to the reference group of complete adenomectomy were: 1.32 (95% CI: 0.57–3.06) for (at least) partial adenomectomy (1.21, 95% CI: 0.43–3.39, for the first 5 years of follow-up), and 3.90 (95% CI: 0.86–17.63) for residual tumor (4.49, 95% CI: 0.96–21.01, for the first 5 years of follow-up). Hazard ratios after adjustment for age at diagnosis, type of adenomectomy (microscopy or endoscopy), and tumor size (microadenoma or macroadenoma) were similar. To assess a potential learning curve within the endoscopic surgery group, patients that underwent endoscopic surgery until December 31st 2011 (*N* = 21) were compared with patients that underwent endoscopic surgery after January 1st 2012 (*N* = 18). To determine whether preoperative imaging quality influenced surgical success in microscopic surgery, patients with CT/X-ray imaging were compared with patients with MRI scans. We found no difference between early and late years of endoscopic surgery (hazard ratio 1.72, 95% CI: 0.46–6.52, which was exactly the same if only the first 5 years of follow-up were included; hazard ratio 1.06, 95% CI: 0.25–4.53 after adjustment), or between preoperative imaging techniques for microscopic surgery (hazard ratio 0.99, 95% CI: 0.36–2.73, which was 0.65, 95% CI: 0.16–2.72, if only the first 5 years of follow-up were included; similar after adjustment), see Fig. 6.

### Overall survival

**Fig. 7:**
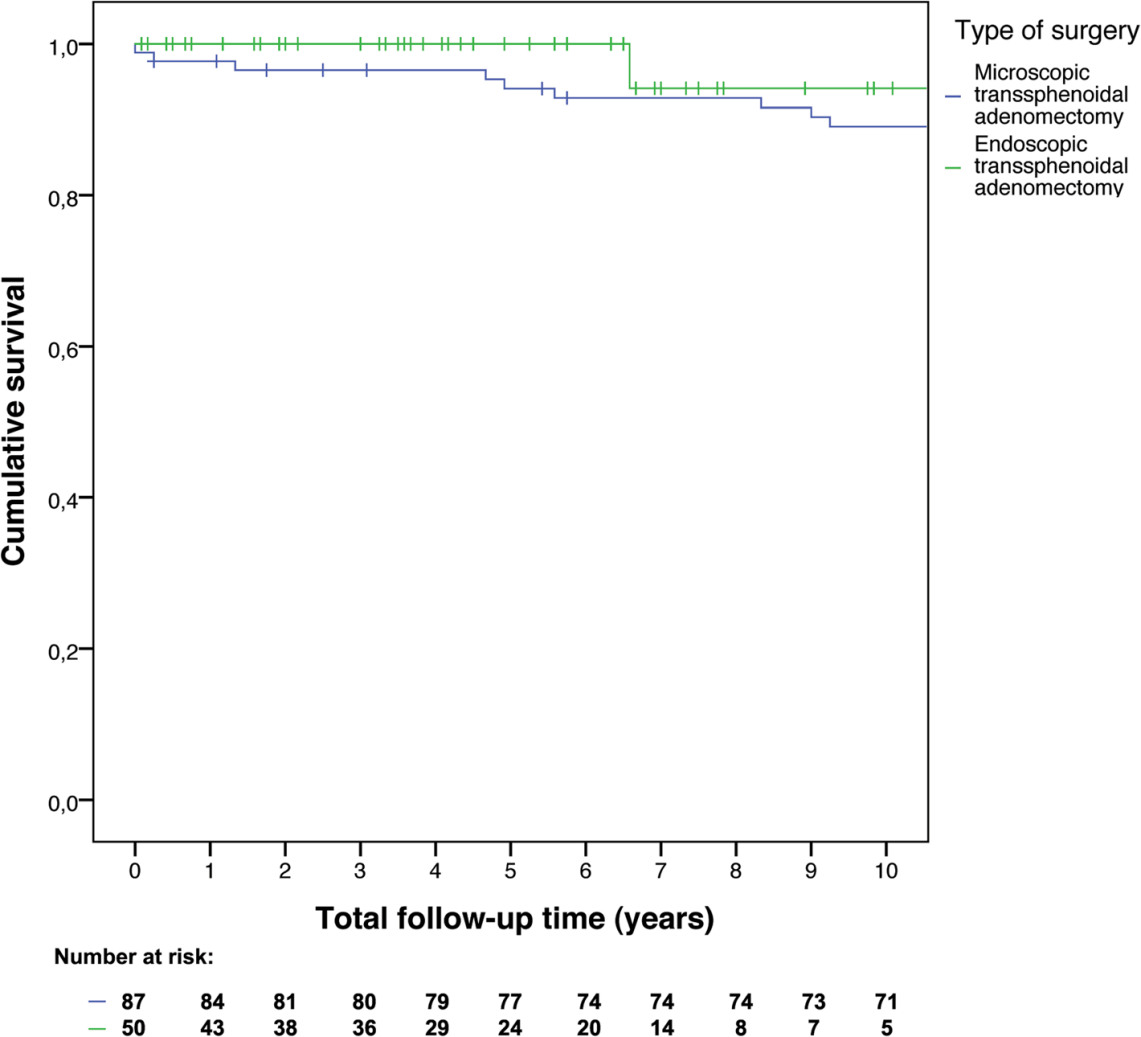
Overall survival after microscopic versus endoscopic transsphenoidal surgery

Within ten years after surgery, ten patients died: nine in the microscopic surgery group and one in the endoscopic surgery group (all-cause mortality). One patient died three days after microscopic surgery of unidentified cause, although a cardiogenic cause was most likely, as left ventricular hypertrophy, coronary artery disease with over 50% stenosis, and pulmonary edema were found at autopsy. Ten-year overall survival rate was 89% (95% CI: 82–96%) for microscopic surgery and 94% (95% CI: 83–100%) for endoscopic surgery. The hazard ratio for mortality was 2.79 (95% CI: 0.35–22.51, similar after adjustment) for microscopic versus endoscopic surgery, see Fig. 7.

### Short- and long-term morbidity

In the microscopic group, less patients experienced a corticosteroid withdrawal syndrome compared to the endoscopic group (hazard ratio after adjustment: 0.38, 95% CI 0.20 to 0.72). Patients with microscopic surgery also had less long-term neuropsychiatric morbidity after surgery than patients with endoscopic surgery. In Table 3, a detailed overview is presented of hazard ratios for short- and long-term morbidity for endoscopic versus microscopic surgery.

Four patients were treated with antibiotics for meningitis, all after microscopic surgery (two of whom had positive CSF cultures, with pseudomonas and klebsiella species, respectively). Three patients had a severe bleeding (two in the microscopy group and one in the endoscopy group), which was discovered directly after surgery based on clinical findings/deterioration and confirmed on postoperative imaging: of these patients one patient had a subdural hematoma (microscopy), one patient had a intracerebral hematoma (microscopy) and a subarachnoid bleeding (both patients were treated surgically), and one patient had severe epistaxis and hematemesis (treated conservatively, but described as severe in the patient file, with at least 800 ml of blood loss; endoscopy). The other, non-severe bleedings were intraoperative venous bleedings (predominantly from the cavernous sinus), that were excessive compared to the expected amount of blood loss during surgery, and another episode of mild epistaxis which was treated conservatively.

### Repeated transsphenoidal surgery

Fifteen patients with initial microscopic surgery underwent a second transsphenoidal surgery after a mean of 111 months (range: 7–242), of which ten were microscopic and five endoscopic. Remission was obtained in five of these patients (33.3%) (all hydrocortisone dependent [absolute deficiency], as evaluated three months after repeated surgery), one of whom had undergone endoscopic and four repeated microscopic surgery. In the endoscopic group, thirteen patients underwent repeated transsphenoidal surgery after a mean of 21 months (range: 1–43), all of which were endoscopic. Remission was obtained in five patients (41.7%) (four hydrocortisone dependent [absolute deficiency] three months after repeated surgery), and duration of follow-up was insufficient in one patient to determine remission status after repeated surgery. A third transsphenoidal surgical procedure was performed in three patients, resulting in remission in two patients (one hydrocortisone dependent [absolute deficiency]). For more detailed information on adjuvant treatment and remission status, see Fig. 1.

## Discussion

In this study, we compared remission rates, mortality risk, and short- and long-term complications between patients treated at a European reference center for Cushing’s disease with microscopic versus endoscopic transsphenoidal pituitary surgery. Both techniques yielded similar results regarding remission rate and percentage of patients with hydrocortisone dependency. Patients with microscopic surgery had longer recurrence-free survival, but a higher mortality rate, than patients with endoscopic surgery. When analysed separately, patients with a macroadenoma had a longer recurrence-free survival after microscopic surgery than after endoscopic surgery. Our results could not prove indications of the presence of a learning curve for endoscopic surgery, nor did we find that the type of imaging technique influenced our results for microscopic surgery. No clear relation was found between the surgeons’ intraoperative findings and recurrence-free survival, although patients with a certain residual tumor after surgery seemed to perform worse than patients with complete adenomectomy. After endoscopic surgery, more patients had symptoms consistent with corticosteroid withdrawal, and patients showed a higher hazard ratio for long-term neuropsychiatric morbidity.

While there have been previous cohort studies, a major strength of this study is that this study includes the largest cohort to date, thereby increasing reliability of results stratified by tumor size. This is also the first cohort study investigating long-term mortality in Cushing’s disease after endoscopic surgery and comparing this to long-term mortality after microscopic surgery. The remission rates and surgical morbidity obtained from this study are in line with previously published small cohort studies in Cushing’s disease directly comparing both techniques [9–12]. Our recently published systematic review comparing both surgical techniques in Cushing’s disease showed an advantage of performing endoscopic surgery for macroadenomas based on remission and recurrence rates [26]. This could not be confirmed in the current cohort study.

There are several study limitations that need to be taken into account when interpreting the study results. Patients treated microscopically were included in a different time period than patients treated by endoscopic surgery, which might have resulted in substantial differences between both study groups. The prevalence of certain comorbidities has changed over time, as well as the available treatment modalities for these comorbidities. However, patient characteristics were compared between the study groups, and only follow-up duration and the use of medical treatment prior to surgery differed largely. It is impossible to say in which direction mortality might have changed due to the differences in prevalence and treatment of comorbidities over time, as one might expect a worse survival with a higher prevalence of comorbidities, but a better survival due to improved treatment modalities. It is unlikely that recurrence-free survival is influenced by prevalence or treatment of comorbidities. Although we adjusted for confounders, residual confounding due to unmeasured or incorrectly measured confounders remains a potential issue. To exclude a potential chronological bias completely, a new study should be performed comparing both surgical techniques in the same study center and in the same time period.

Selective loss to follow-up could have led to selection bias, as all patients lost to follow-up were in the microscopic surgery group. As patients could have become lost to follow-up due to various reasons (including both excellent health as well as very poor health status), the direction in which the results may have been biased cannot be determined. This effect was minimized as our analyses were confined to the first ten years after surgery and loss to follow-up was on average 8.3 years after surgery. Selection due to selective referral for endoscopic surgery to our center of mainly complex cases (i.e. macroadenomas and/or those with inconclusive imaging features suggesting lateral invasion of the cavernous sinus), may have led to worse surgical outcomes and higher complication rates after endoscopic surgery than expected. This is exemplified by the higher percentage of adenomas with cavernous sinus invasion in the endoscopically treated group.

Over time, diagnostic strategies and treatment for Cushing’s disease have changed. Diagnostic imaging has changed from CT to MRI resulting in better visualization of suspect lesions, increasing the certainty of which target to address during surgery. There have been several treatment changes at our center, e.g. the surgical technique has changed from microscopic surgery to endoscopic surgery, and after 1990, preoperative medical treatment to control cortisol secretion became common practice at our center. Patients were not randomized to a certain treatment but were treated by the method available at that time. Therefore, effects of time, imaging technique, and use of medical therapy prior to surgery, cannot be separated from the effect of the surgical technique. To evaluate the effect of preoperative medical treatment on outcome per se, a randomized trial is needed which compares patients with the same surgical technique with and without medical pretreatment, which has not yet been performed to our knowledge.

According to the STROBE statement, generalizability of the study needs to be discussed [25]. Theoretically, this study is generalizable to all Cushing’s disease patients with a primary surgical treatment of transsphenoidal adenomectomy. However, generalizability is reduced due to specific effects produced by the setting (hospital, neurosurgeons) and time period of inclusion of patients per study group.

In the endoscopic group, a higher rate of corticosteroid withdrawal syndrome was seen. This is especially remarkable, as many patients received preoperative medical treatment to lower cortisol levels before endoscopic surgery. However, this result is in agreement with our clinical experience, and might be explained by increased awareness for the corticosteroid withdrawal syndrome during recent years, leading to better recognition and improved hydrocortisone adjustments, when needed. Patients treated endoscopically showed a higher hazard ratio for long-term neuropsychiatric morbidity. This is, at least in part, due to the increased awareness of persistent neuropsychiatric comorbidity in patients with (previous) Cushing’s disease in recent years.

Better results were anticipated for endoscopic surgery compared to microscopic surgery regarding recurrence-free survival and complication rates, based on the better visualization of tumors, certainly for the laterally invasive or large tumors. Regarding recurrence-free survival, we found an advantage of microscopic surgery for macroadenomas only. Microadenomas are most likely completely within the field of vision regardless of the surgical technique, explaining the lack of difference between both surgical techniques. As our recently published review showed an advantage of endoscopic surgery for macroadenomas [26], the advantage of microscopic surgery for macroadenomas shown in this study regarding recurrence-free survival might be explained by the surgeons’ attempt to perform a complete tumor resection with limited visibility of the entire tumor, although extent of anterior pituitary deficiency after surgery was similar for both techniques. Another explanation is that more difficult and invasive macroadenomas were referred to our center for endoscopic surgery (six endoscopic versus four microscopic treated patients were referred by other neurosurgeons or university medical centers), resulting in eight endoscopic (50.0%) versus five microscopic (27.8%) macroadenomas with cavernous sinus invasion. However, excluding patients with macroadenomas and cavernous sinus invasion did not change the hazard ratio for recurrence. Differences between both surgical techniques may also have occurred due to the small number of patients per study group after stratification by tumor size. Differences in outcomes between the current cohort study and our recently published systematic review and meta-analysis on the same subject [26] may have resulted from differences in included patients in both studies, e.g. as a result of selective referral of more difficult and invasive macroadenomas to our center, or as a result of differences in experience in and use of both techniques for Cushing’s disease or also other pituitary adenomas between our center and other centers included in the systematic review. Differences may also have resulted from variations in used definitions for remission, recurrence, and complications between study centers. The difference in outcome between the current cohort study and our recently published systematic review emphasizes the importance of combining data from multiple centers, as will be done for patient care purposes in the newly established European Reference Network on rare endocrine conditions (Endo-ERN).

## Conclusions

We found no clear advantage for either microscopic or endoscopic transsphenoidal surgery in Cushing’s disease, based on surgical outcome or complications. In addition, no learning curve was found for endoscopic surgery. The transition from microscopic surgery to endoscopic surgery for Cushing’s disease in our center was made without temporarily deterioration of outcomes, which may be reassuring for surgeons who consider changing to endoscopic surgery.
